# Mitigating Methylmercury Exposure: Study Confirms Potential of NAC as Antidote and Biomarker

**DOI:** 10.1289/ehp.116-a36a

**Published:** 2008-01

**Authors:** Kris Freeman

Researchers have been searching for better ways to quantify and mitigate exposures to the neurotoxicant methylmercury (MeHg). Results from a new animal study confirm that *N*-acetylcysteine (NAC), already used to treat acetaminophen overdose, may serve as a quick-acting antidote for and biomarker of MeHg exposure **[*EHP* 116:26–31; Aremu et al.]**.

MeHg is created when elemental mercury released through the burning of coal, waste incineration, and other industrial processes is metabolized by aquatic microorganisms such as anaerobic bacteria. It bioaccumulates rapidly, with concentrations in some top marine predators reaching 100,000 times that of surrounding seawater. Fish consumption is the major source of human exposure. MeHg can cause irreversible brain damage, and the developing brain is especially vulnerable to its effects.

Treatments to mitigate MeHg exposure involve chelation, the administration of compounds that bind mercury, speeding its elimination from the body and thereby minimizing its toxicity. Current chelation methods can be nonspecific, depleting not only MeHg but also minerals required for normal cell function, such as calcium.

In contrast, chelation treatment with NAC does not affect levels of essential minerals. NAC, a derivative of the amino acid l-cysteine and a precursor of the antioxidant glutathione, is itself a potent antioxidant. NAC can be delivered intravenously or orally.

In the current study, adult rats were injected with NAC (1 mmol/kg) 2 hours after being exposed to MeHg (0.1 μmol/kg). The treated animals excreted about 5% of their body burden of MeHg within 2 hours, compared with less than 0.1% excreted by untreated animals. The response was transient and dose dependent, with larger doses of NAC resulting in higher rates of MeHg excretion.

These effects were not seen in preweaned rats (age 15–19 days) treated with NAC. The researchers speculate that the transporter systems needed to move the MeHg–NAC complex through the kidney do not mature until animals reach adulthood (around 30 days of age). However, oral NAC treatment in pregnant rats (10 mg/mL in drinking water) did protect their fetuses, reducing concentrations in the placenta and the whole fetus by 70–90%. In the dams themselves, NAC also reduced MeHg concentrations by 70–90% in the brain, by about 20% in the kidney, and by 60–80% in the blood and liver.

NAC’s short half-life, about 2 hours, may allow it to serve as an accurate real-time biomarker of MeHg exposure. According to the researchers, such a quick-acting biomarker could provide critical early warning of possible acute exposures, where early treatment is critical to prevent neurological damage. In the current study, MeHg excretion in animals treated with NAC was proportionate to MeHg body burden at the time of treatment. In contrast, standard monitoring techniques, which use hair analysis, can provide only a history of exposure and cannot guide immediate treatment for acute exposures. The researchers propose that future studies test NAC in adult humans as a biomarker of exposure and a possible treatment for MeHg exposure, especially for pregnant women whose unborn children are in danger of prenatal MeHg exposure.

## Figures and Tables

**Figure:**
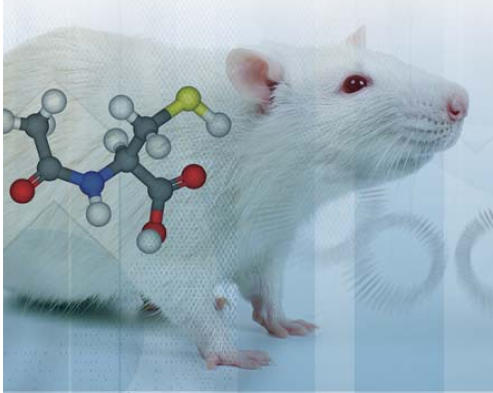
NAC offers promise as an antidote to acute MeHg exposure

